# Detection of Urinary Tract Pathology in Some *Schistosoma haematobium* Infected Nigerian Adults

**DOI:** 10.1155/2016/5405207

**Published:** 2016-08-22

**Authors:** O. S. Onile, H. O. Awobode, V. S. Oladele, A. M. Agunloye, C. I. Anumudu

**Affiliations:** ^1^Department of Biological Sciences, Elizade University, P.M.B. 002, Ilara-Mokin, Ondo State, Nigeria; ^2^Parasitology Unit Department of Zoology, University of Ibadan, Ibadan 20004, Nigeria; ^3^Cellular Parasitology Programme, Department of Zoology, University of Ibadan, Ibadan 200004, Nigeria; ^4^Department of Radiology, University Teaching Hospital, University of Ibadan, Ibadan 200004, Nigeria

## Abstract

Screening for* Schistosoma haematobium* infection and its possible morbidity was carried out in 257 adult participants in Eggua community, Ogun State, Nigeria. Parasitological assessment for the presence of ova of* S. haematobium* in urine and abdominopelvic ultrasonographic examination for bladder and secondary kidney pathology were carried out.* S. haematobium* prevalence of 25.68% (66/257) was recorded among the participants. There was a significantly higher prevalence of 69.2% of urinary schistosomiasis in the females than the prevalence of 31.8% in males (*P* = 0.902). The intensity of infections was mostly light (55) (21.8%) compared to heavy (10) (3.9%) with the mean intensity of 16.7 eggs/10 mL urine. Structural bladder pathology prevalence among participants was 33.9%. The bladder and kidney pathologies observed by ultrasound in subjects with* S. haematobium* infections included abnormal bladder wall thickness (59%), abnormal bladder shape (15.2%), bladder wall irregularities (15.2%), bladder masses (1.5%), bladder calcification (1.5%), and hydronephrosis (3%). Infection with* S. haematobium* was associated with bladder pathology. Higher frequencies of bladder abnormalities were observed more in the participants with light intensity of* S. haematobium* infection than in those with heavy infection. More bladder pathology was also seen in women than in men, although this was not statistically significant. In conclusion, there is evidence that the development of bladder pathology may be associated with *S. haematobium* infection.

## 1. Introduction

An estimated 207 million cases of human schistosomiasis have been reported worldwide and about 90% of these live in Sub-Saharan Africa, with Nigeria having the highest prevalence [1].* Schistosoma* infections cause significant morbidity and mortality with peak prevalence and intensity of infection occurring between the ages 10 and 20 years and subsequent decline by age 65 years [2].

Chronic human circulatory system infection by* Schistosoma haematobium* is reported to affect the urinary bladder and is a possible risk factor in the aetiology of cancers of the bladder and the urinary tract system [3].* S. haematobium* infection has been linked with the development of squamous cell carcinoma of the bladder [4, 5].* S. haematobium* associated bladder damage has been closely linked to the immune reaction elicited against the parasite egg deposited in the bladder which eventually induces chronic inflammation related granulomatous injury [6].

Schistosomiasis and bladder cancer share common symptoms such as haematuria, dysuria, and pain with micturition. This may prevent early diagnosis of bladder cancer and the resultant severe bladder damage particularly in people living in* S. haematobium* endemic areas.

In Nigeria, most studies have focused on the epidemiology of* S. haematobium* infection [3, 7, 8] particularly in school-age children, with limited information about the morbidity resulting from urinary schistosomiasis in adults.

This study was therefore aimed at determining the prevalence of schistosomiasis and associated bladder pathology in adults living in Eggua, Yewa, North Local Government Area, Ogun State, Nigeria.

## 2. Materials and Methods

The study was carried out in Eggua, a rural agrarian community, between August 2012 and May 2013. It is one of the wards that make up Yewa North Local Government Area as previously described [9]. Eggua lies between latitude 7°6′4.811′′N and longitude 2°52′43.776′′E in a derived savanna zone. The area is largely dominated by Yoruba speaking people. It consists of settlements at Sagbon, Imoto, Tata, Agbon-Ojodu, and Igan Alade. It shares boundaries with Igbogila, Ilaro, Ijoun, and Benin Republic.

Two major rivers (Yewa and Iju) flowing through the area serve as the main water source, resulting in high water contact by the inhabitants. These rivers are used for religious, domestic, and entertainment activities which enhance the transmission of schistosomiasis.

A cross-sectional study design was employed for this study. Participants aged 30 to more than 60 years old from the community were enrolled for the study. Children were excluded from the study in line with the objective of the study to determine the effect of chronic urinary schistosomiasis on adult members of the community.

### 2.1. Ethical Considerations

Informed consent was obtained from each participant under a protocol approved by the Local Government and local health officials. Ethical approval for the study was also obtained from the Ogun State Ministry of Health.

### 2.2. Sample (Biofluid) Collection

Blood (5 mL) and urine specimens were collected from each study participant. The urine samples were collected between 10:00 and 14:00 hours to ensure maximum egg yield. Packed cell volume (PCV) was determined from the blood collected.

### 2.3. Sample Analyses

The urine samples (10 mL) were processed for microscopic examination and egg count [3, 10]. The eggs were quantified by counting under the microscope and classified as light infection if there were ≤50 (1–49) eggs/10 mL urine and heavy infection if there were >50 eggs/10 mL urine [3].

### 2.4. Ultrasound and Pathology

A blind ultrasound examination was carried out on each participant approximately 1 h after drinking potable water (0.1–1.5 litre depending on the age of the participant) to distend the bladder. The classification of bladder pathology or damage was based on the definition of the WHO [11, 12] and Shiff et al. [5]; the abnormalities assessed included abnormal bladder shape, bladder wall irregularities, bladder masses, presence of polyps, calcification, and presence of hydronephrosis in the kidneys. Bladder lesions were considered severely abnormal when four of the above conditions or three conditions as well as hydronephrosis were present in a single individual. Lesions were considered moderate if fewer conditions were seen and negative when no specific lesions were observed.

### 2.5. Sociodemographic Data Collection

A structured, pretested questionnaire was used to obtain information about participants' habits regarding smoking and alcohol consumption, which are determinants of bladder cancer. Sociodemographic information was also recorded for each of the participants.

Statistical analysis of data obtained was done using SPSS version 20.0 (*P* < 0.05).

## 3. Results

A total of 257 (79 males and 178 females) participants aged 30–90 years were screened for* S. haematobium* infection and associated bladder pathologies. The mean age of participants was 48 ± 12.2 years. The overall prevalence of* S. haematobium* in the sampled population was 25.68% (66/257), 21 (31.8%) in males and 45 (68.2%) in females. The highest prevalence of infection was observed in participants over 60 years old (Table 1). The majority (56/66) (84.8%) of those positive for* S. haematobium* had a light intensity of infection with the egg mean intensity of 16.7 eggs/10 mL urine. The Yewa river was the main source of water for most (49/62) (79.0%) of the participants infected with* S. haematobium* (Table 4).

**Figure 1:**
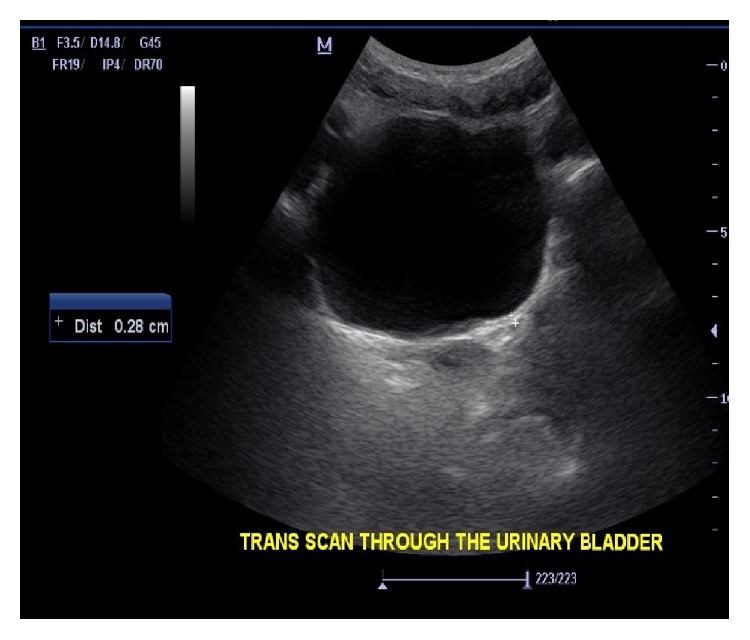
B mode ultrasound of the bladder showing a fully extended bladder with no pathology.

**Figure 2 fig2:**
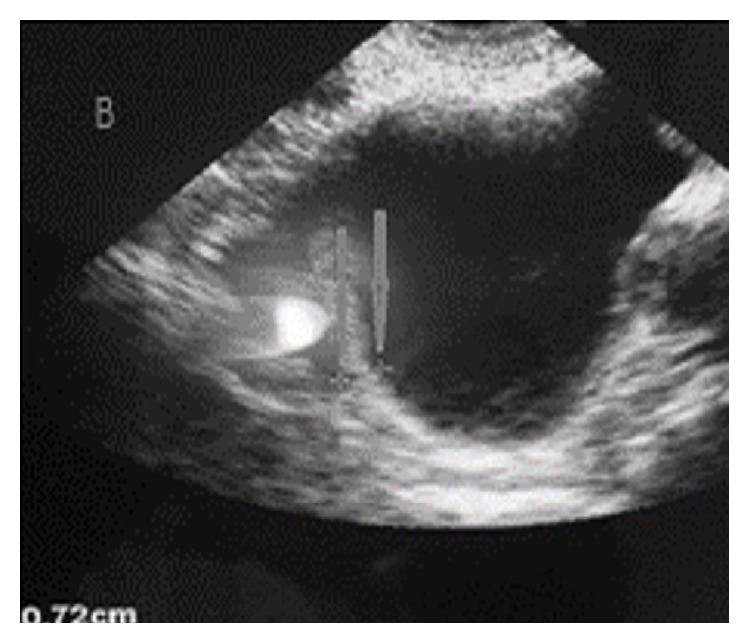
B mode ultrasound of the bladder showing a thickened bladder wall (arrows).

**Figure 3:**
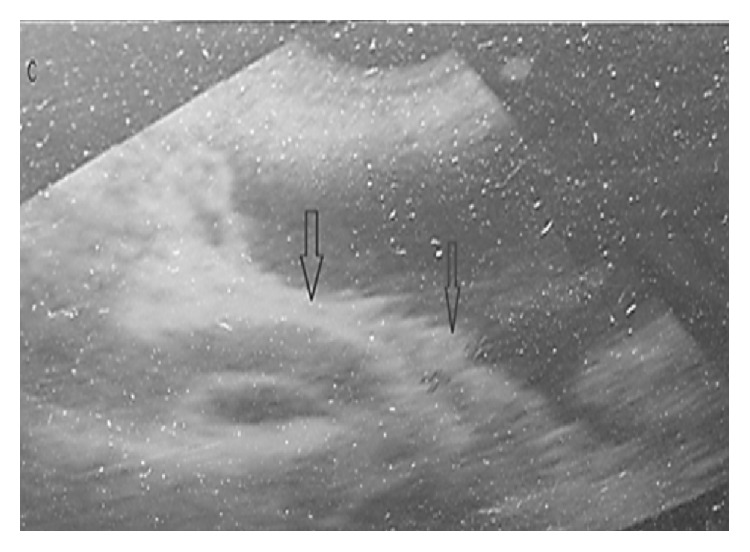
Bladder with masses extending from the wall into the lumen (ignore speckled artefacts).

Bladder pathologies were observed in 33.9% (87/257) of the sample population and included abnormal bladder wall thickness (39/66) (59%), abnormal bladder shape (10/66) (15.2%), bladder wall irregularities (15.2%), bladder masses (1.5%), and bladder calcification (1.5%) (Table 2). Bladder wall thickness, the most common abnormality, was recorded in 46/79 (58.2%) males and 90/178 (50.6%) females (Table 3). Among the participants, 56 (84.8%) with bladder pathologies also had an existing schistosomiasis infection, 48 (87.3%) of which were light intensity and 8 (72.7%) of which were heavy intensity: *χ* ^2^ = 267.5, *P* = 0.001 (Table 5). Thus, there was an association between urinary tract pathology and the intensity of* S. haematobium* infection (*χ* ^2^ = 375.4, *P* = 0.001, Table 2). Among the participants with light and heavy intensity of* S. haematobium* infections, bladder wall thickness was the most common bladder structural pathology identified in 33/56 (58.9%) and 6 (60.0%) participants with light and heavy* S. haematobium* infections, respectively (Table 5). Abnormal bladder shape and bladder wall irregularity were seen in 8/56 (14.3%) and 2 (20%) participants with light and heavy infections, respectively (Figures 1 [Fig fig2]–3). Hydronephrosis was present in only one participant with light infection, while calcification was identified in only one participant with heavy infection. No bladder polyp was detected. Mild bladder pathology was more common than severe bladder pathology in this study and was found in 48 of the participants (Table 5). There was a higher incidence of bladder pathologies among female participants (Table 3); bladder mass and hydronephrosis were also seen only in female participants.

There was no significant relationship between cigarette smoking and bladder pathology in the study (Table 6). Among participants with bladder pathology, 29 (33.3%) admitted consuming alcohol while 58 (66.7%) said that they had never consumed alcohol (Table 7).

## 4. Discussion

The overall prevalence rate (25.98%) of adults with* S. haematobium* infection recorded in this study was slightly higher than 20.8% and 20.0% reported in Yewa North Local Government, Ogun State, and Owan East Local Government, respectively, in Nigeria [3, 13].

Most (81.3%) of the participants depended solely on the* S. haematobium* contaminated river water, which could account for the higher* S. haematobium* prevalence; and little or no schistosomiasis control (drug) intervention targeted to adults has been recorded in this area. The higher frequency of light intensity* S. haematobium* infection observed in this study could be explained by some level of acquired protected immunity by adults in that community due to chronic exposure to schistosomiasis. Shiff et al. [5] found that the proportion of egg-positive individuals falls progressively with age and is a feature in populations with lifelong exposure to the parasite. Therefore, chronicity of infections in older people will more likely be difficult to ascertain using egg count method. The higher frequency of mild bladder pathology observed in this study was also similar to another study [14] which observed a higher incidence of mild bladder than severe bladder pathology. This result could be explained by the low number of participants who smoked cigarettes and consumed alcohol; these conditions may serve as promoting factors either in progression of bladder pathology to cancer or in making the bladder pathology more severe (Table 4). In addition, this lifestyle could buttress the possibility of* S. haematobium* being the principal cause of the reported bladder structural pathology in the study population.

The close relationship between the intensity of* S. haematobium* infection and the presence of bladder abnormalities was similar to previous reports [3, 14–16]. The presence of hydronephrosis in participants with light infection is however at variance with the report of Nmorsi et al. [3] although hydrocalycosis (a condition mostly mistaken for hydronephrosis) was observed in some patients with heavy infection, indicating the likely contribution of this infection to kidney pathology. Females (64.7%) had more structural bladder pathology compared to males (35.3%). This may be due to higher water contact by females and also to the higher number of female study participants than an indication of a female predilection to bladder pathology. However, since hydronephrosis and bladder mass or bladder calculi were found together in a female participant, female predilection to bladder pathology may not completely be ruled out. The structural changes to the bladder recorded in this study were in consonance with observations in West Madagascar [14] and Nigeria [3, 15] where bladder irregularities and bladder wall thickness were identified as the most common pathologies in individuals infected with* S. haematobium*.

In conclusion, there is evidence that* S. haematobium* infections may be associated with bladder pathology, on ultrasound examination. Individuals with bladder pathologies could have heavy or light intensity of schistosomiasis infection or have no existing infection at all. However, a long term exposure to schistosomiasis is necessary for the development of bladder cancer. Further research on the determinants and progress of the bladder pathologies seen in this study population is needed.

## Figures and Tables

**Table 1:** Prevalence and intensity of *S. haematobium* by sex and age group in Eggua, Nigeria.

	Light *N*≤ 50 (%)	Heavy *N* > 50 (%)	Total
Sex			
Male	18 (27.3)	3 (4.5)	21 (31.8)
Female	38 (57.6)	7 (10.6)	45 (69.2)

Total	**56 (21.8)**	**10 (3.9)**	**66 (25.68)**

*χ* ^2^ = 2.514, *P* = 0.113			

Age group			
30–34	2 (3.6)	1 (10)	
35–39	9 (16.1)	1 (10)	
40–44	8 (14.3)	1 (10)	
45–49	9 (16.1)	1 (10)	
50–54	6 (10.7)	2 (20)	
55–59	6 (10.7)	2 (20)	
60 and above	16 (28.6)	2 (20)	

**Table 2:** Distribution of bladder pathology with intensity of *S. haematobium* infection.

Pathology *χ* ^2^ = 375.4, *P* = 0.001	Intensity of infection		Total
	Light (%)	Heavy (%)	
Bladder wall thickness	33 (58.9)	6 (60.0)	59%
Bladder shape	8 (14.3)	2 (20.0)	15.2%
Bladder wall irregularity	8 (14.3)	2 (20.0)	15.2%
Bladder mass	0 (0)	1 (10.0)	1.5%
Calcification	0 (0)	1 (10.0)	1.5%
Polyps	—	—	—
Hydronephrosis	6 (10.7)	0 (0)	9%

**Table 3:** Distribution of bladder pathology among genders.

Pathology	Gender	
	Male	Female
Bladder wall thickness	46 (58.2)	90 (50.6)
Bladder shape	7 (8.9)	6 (3.4)
Bladder wall irregularity	7 (8.9)	6 (3.4)
Bladder mass	—	1 (0.6)
Calcification	—	1 (0.6)
Polyps	—	—
Hydronephrosis	—	6 (3.4)

**Table 4:** Relative risk estimates of schistosomiasis with sources of water.

Sources of water use *χ* ^2^ = 4.789, df = 2, *P* = 0.091	Status of *S. haematobium* infection		Total
	Positive	Negative	
Rivers	49 (79.0)	160 (88.9)	209 (84.3)
Others	11 (17.7)	15 (8.1)	26 (10.5)
Rivers and others	2 (3.2)	11 (5.9)	13 (5.2)

Total	62 (100.0)	186	248

**Table 5:** Relation between intensity of bladder pathologies and intensity of *S. haematobium* infection.

Intensity of *S. haematobium* infection *χ* ^2^ = 267.5, *P* = 0.001	Intensity of bladder pathology		Total *N* (%)
	Mild *N* (% )	Severe *N* (%)	
Intensity of infection			
Heavy	6 (9.09)	2 (3.0)	8 (12.1)
Light	42 (63.63)	6 (9.1)	48 (72.7)

Total	48 (69.69)	8 (12.1)	56 (84.8)

**Table 6:** Relative risk estimates of bladder pathology associated with cigarette smoking.

Cigarette smoking *χ* ^2^ = 0.67, *P* = 0.880	Pathology		Total *N* (%)
	Present *N* (%)	Absent *N* (%)	
No response	5 (5.7)	12 (7.1)	17 (5.8)
Yes	4 (4.6)	5 (2.9)	9 (12.1)
No	78 (89.7)	153 (90.0)	231 (82.1)

Total	**87 (33.9)**	170 (66.1)	257

**Table 7:** Relative risk estimates of bladder pathology associated with alcohol consumption.

Alcohol consumption *χ* ^2^ = 3.549, *P* = 0.170	Bladder pathology		Total
	Present	Absent	
Yes	29 (33.3)	72 (42.4)	101 (39.3)
No	58 (66.7)	85 (50)	143 (55.6)
No response	0 (5.7)	13 (7.6)	13 (5.1)

## References

[1] Ugbomoiko U. S., Ofoezie I. E., Okoye I. C., Heukelbach J. (2010). Factors associated with urinary schistosomiasis in two peri-urban communities in south-western Nigeria. *Annals of Tropical Medicine & Parasitology*.

[2] Rambau P. F., Chalya P. L., Jackson K. (2013). Schistosomiasis and urinary bladder cancer in North Western Tanzania: a retrospective review of 185 patients. *Infectious Agents and Cancer*.

[3] Nmorsi O. P. G., Ukwandu N. C. D., Ogoinja S., Blackie H. O. T., Odike M. A. C. (2007). Urinary tract pathology in some Schistosoma haematobium infected Nigerians. *African Journal of Biotechnology*.

[4] Botelho M. C., Machado J. C., da Costa J. M. C. (2010). Schistosoma haematobium and bladder cancer: what lies beneath?. *Virulence*.

[5] Shiff C., Veltri R., Naples J. (2006). Ultrasound verification of bladder damage is associated with known biomarkers of bladder cancer in adults chronically infected with Schistosoma haematobium in Ghana. *Transactions of the Royal Society of Tropical Medicine and Hygiene*.

[6] Hotez P. J., Brindley P. J., Bethony J. M., King C. H., Pearce E. J., Jacobson J. (2008). Helminth infections: the great neglected tropical diseases. *Journal of Clinical Investigation*.

[7] Agere I. J., Istifanus W. A., Kela S. L. (2010). Water usage and transmission of *Schistosoma haematobium* in jalingo and ardokola local governmentareas of Taraba State, Nigeria. *Nigerian Journal of Science, Technology and Environmental Education (NIJOSTEE)*.

[8] Agbolade O. M., Odaibo A. (1996). *Schistosoma haematobium* infection among pupils, and snail intermediate hosts in Ago-Iwoye, Ogun State. *The Nigerian Journal of Parasitology*.

[9] Hassan A., Uduak N., Morenikeji O. (2012). Urine turbidity and microhaematuria as rapid assessment indicators for schistosoma haematobium infection among school children in endemic areas. *American Journal of Infectious Diseases*.

[10] Weber M. D., Blair D. M., Clark V. V. (1967). The pattern of schistosome egg distribution in a micturition flow. *Central African Journal of Medicine*.

[11] WHO (1996). Meeting on ultrasonography in schistosomiasis: proposal for a practical guide to the standardized use of ultrasound in assessment of pathological changes. *TDR/SCH/Ultrason*.

[12] World Health Organization (WHO) (2000). Ultrasound in schistosomiasis. A practical guide to the standardized use of ultrasonography for assessment of schistosomiasis-related morbidity.

[13] Salawu O. T., Odaibo A. B. (2013). Schistosomiasis among pregnant women in rural communities in Nigeria. *International Journal of Gynecology and Obstetrics*.

[14] Serieye J., Boisier P., Ravaoalimalala V. E. (1996). Schistosoma haematobium infection in western Madagascar: morbidity determined by ultrasonography. *Transactions of the Royal Society of Tropical Medicine and Hygiene*.

[15] Ekwunife C. A., Okafor F. C., Nwaorgu O. C. (2009). Ultrasonographic screening of urinary schistosomiasis infected patients in Agulu community, Anambra state, southeast Nigeria. *International Archives of Medicine*.

[16] Warren K. S., Mahmoud A. A. F., Muruka J. F., Whittaker L. R., Ouma J. H., Arap Siongok T. K. (1979). Schistosomiasis haematobia in Coast Province Kenya. Relationship between egg output and morbidity. *American Journal of Tropical Medicine and Hygiene*.

